# Nicotine Increases Cerebellar Activity during Finger Tapping

**DOI:** 10.1371/journal.pone.0084581

**Published:** 2013-12-17

**Authors:** Korey P. Wylie, Jody Tanabe, Laura F. Martin, Narin Wongngamnit, Jason R. Tregellas

**Affiliations:** 1 Department of Psychiatry, University of Colorado Anschutz Medical Campus, Aurora, Colorado, United States of America; 2 Department of Radiology, University of Colorado Anschutz Medical Campus, Aurora, Colorado, United States of America; 3 Research Service, Denver Veterans Affairs Medical Center, Research Service (151), Eastern Colorado Health System, Denver, Colorado, United States of America; Peking University, China

## Abstract

Nicotine improves performance on several cognitive and sensorimotor tasks. The neuronal mechanisms associated with these changes in performance are, however, largely unknown. Functional magnetic resonance imaging (fMRI) was used to examine the effect of nicotine on neuronal response in nineteen healthy subjects while they performed an auditory-paced finger tapping task. Subjects performed the task, after receiving either a nicotine patch or placebo treatment, in a single blind, crossover design. Compared to placebo, nicotine treatment increased response in the cerebellar vermis. Increased vermal activity, in the absence of changes in other task-related regions suggests specificity in nicotine’s effects.

## Introduction

In order to better understand nicotine’s effects on brain function, it is useful to examine its effects during behaviors known to be affected by nicotine. Nicotine’s performance-improving effects on simple motor tasks have been well documented [1]. One of the most commonly studied motor tasks is finger tapping. It is both abnormal in many disease states and improved by nicotine administration [2,3]. In healthy non-smokers, nicotine administration is associated with an increase in unpaced tapping rate [4]. The functional neuroanatomy of this task has been well established, including the primary sensorimotor cortex, supplementary motor area, and cerebellum [5-7]. Despite its ability to improve performance during this task, nicotine’s effect on neuronal response during finger tapping tasks has not been investigated.

On the molecular level, nicotine acts on ionotropic nicotinic acetylcholine receptors (nAChR) within the brain. These receptors are found widely throughout the cerebrum and cerebellum [8,9]. The diffuse distribution of nAChRs leads to neuromodulatory effects of nicotine on many cognitive systems within the brain. The nicotinic cholinergic system is involved in many psychomotor and cognitive functions, including sensory gating [10], attention [11], response inhibition [12], and motor coordination [4]. These functions are disrupted in many psychiatric and neurodegenerative diseases and are improved with nicotine [2,3,10]. How nicotine’s interaction with the cholinergic system leads to coordination improvements in healthy subjects and patients is currently a question largely uninvestigated.

To investigate the effect of nicotine on a simple motor task, we used fMRI to measure neuronal response in healthy subjects undergoing an auditory-paced finger tapping task, while receiving either a nicotine patch or placebo, in a single-blind, cross-over, counter-balanced design. We hypothesized that nicotine would enhance responses in areas associated with the task. In particular, frontal and cerebellar areas involved in motor coordination were predicted to be increased by nicotine, as compared to placebo.

## Materials and Methods

### Subjects

Twenty-five right-handed healthy adults participated in this study. Four subjects withdrew due to nausea during the pre-study nicotine tolerance session, one moved out of state, and one subject did not complete all scanning sessions. Data from the remaining 19 subjects (11 male and 8 female, average age of 30 years, SD 9) are reported on here. Three subjects were former smokers, one with a lifetime use of 100 cigarettes and abstinent for 3 years prior to this study. One previously smoked for 2-3 months and had been abstinent for 22 years. One had a lifetime use of 20 cigarettes and was abstinent for 3 years. Three had smoked five or fewer cigarettes in their lifetime. All subjects had been nicotine-free for at least three years. Subjects were excluded for axis I disorders, neurologic illness, or major medical illness. This study was approved by the Colorado Multiple Institutions Review Board, the institutional review board for the University of Colorado at Denver. All subjects provided written consent.

### Experimental Design and Nicotine Administration

A single-blinded, placebo-controlled, crossover design was used. Subjects participated in three sessions. During the first session, medical, psychiatric and smoking histories were obtained and subjects underwent a nicotine tolerance test. During the second and third sessions, subjects were scanned before and after receiving either a 7 mg transdermal nicotine patch (Nicoderm CQ, Alza Corp) covered in tape or the placebo treatment, which included the same tape with no patch. Patches were applied by the study’s physician (LM) and with the subjects eyes closed. Patches were placed on the subject’s back to disguise visual clues about the patch’s identity. Subjects were re-scanned 90 minutes after receiving the patch or placebo, when nicotine levels from the transdermal patch were expected to be near peak [13]. The average time between nicotine and placebo patch sessions was 18.2 days, with the ordering of patches counterbalanced across subjects. After scanning, all subjects were asked if they could tell which patch they had received. No distinguishable difference in their responses after either patch condition was observed, suggesting that the placebo was effective.

### fMRI Data Acquisition and Preprocessing

Images were acquired with a 3T whole-body MR scanner (General Electric, Milwaukee, WI, USA) using standard quadrature head coil. A high-resolution 3D T1-weighted anatomical scan was collected. Functional scans were acquired with the following parameters: TR 1800 ms, TE 32 ms, FOV 240 mm^2^, matrix size 64 x 64, voxel size 3.75 x 3.75 mm^2^, slice thickness 3 mm, gap 0.5 mm, interleaved, flip angle 70 degrees. All data was preprocessed using SPM8 (Wellcome Dept. of Imaging Neuroscience, London, UK) running on Matlab R2012a. The first four images were excluded to allow for saturation effects. Images were realigned to the first volume, normalized to Montreal Neurological Institute (MNI) space using unified segmentation [14], and spatially smoothed with an 8 mm FWHM Gaussian kernel. Translational and rotational movement parameters were less than 3 mm or 3 degrees respectively in any direction for all subjects.

### Finger Tapping Task

Subjects were scanned while pressing a button with their right hand in response to an auditory cue at a constant rate of 1, 2 or 4 Hz. The auditory cue used was a single tone of 250 Hz, with 250 ms duration, and a 70 dB volume level. Since the degree of hemodynamic response in some areas is related to tapping rate [15], a range of rates was used. Individual blocks consisted of 20 seconds for each tapping frequency in a pseudo-randomized order, followed by 20 seconds of rest. An entire run consisted of 5 repetitions of each frequency and the rest condition.

### Data Analysis

Subject’s finger tapping rates were compared to auditory cue rates by calculating inter-tap intervals and entering the results into a one-way repeated measures ANOVA. Statistical analysis of fMRI images was performed in SPM8. During first-level single-subject general linear model analysis, a boxcar function of the block onset and duration for each finger tapping frequency condition was convolved with the canonical hemodynamic response function. A high-pass filter of 256 Hz and a correction AR(1) error model for serial correlations were used. Subject contrast images were constructed by comparing the estimated hemodynamic response over all blocks of tapping frequencies to an implicit rest baseline. The treatment (nicotine or placebo) by session (pre or post scan) interaction was evaluated with directional contrasts (i.e., SPM t-contrasts) in a 2 x 2 repeated-measures ANOVA. Four finger-tapping related ROIs, including left primary motor cortex (PMC), left supplementary motor area (SMA), right anterior cerebellum, and anterior vermal area were examined. The ROIs were chosen based on their in involvement in similar finger-tapping tasks in previous studies [5-7]. The anatomically-based ROIs were created using the templates included in the AAL distribution [16,17]. Results from these areas were corrected for multiple comparisons using a small-volume correction (FDR) and reported at p < 0.05, corrected. The main effect of task across all conditions was evaluated at a more stringent whole-brain voxel-wise threshold of p < 0.05, family-wise error (FWE) corrected. All t-values reported in figures and tables are the directional t-contrasts that reflect the interaction term from the 2 x 2 repeated-measures ANOVA. 

## Results

### Behavioral Results

Reaction times from all experimental conditions and tapping frequencies are shown in Table 1. Inter-response intervals (IRIs) that were less than 50% of the target interval duration were excluded as artifacts. These typically occurred at the beginning of a trial, as the subject started tapping, or adjusted to a new tapping frequency. In all cases, mean IRIs were within 5% of the target interval duration. As expected from task design, no effect of nicotine treatment at any tapping frequency was observed on IRIs (Table 1), which allowed examination of nicotine’s effect on regional neural responses independent of changes in tapping rate.

**Table 1:** Finger tapping frequencies across all experimental conditions.

	Finger Tapping Frequency (ms):		
	1 Hz	2 Hz	4 Hz
Pre-placebo	959.1 (125.70)	489.18 (57.18)	238.59 (22.94)
Post-placebo	958.87 (127.18)	489.01 (54.51)	237.89 (23.26)
Pre-nicotine	962.55 (133.74)	490.32 (57.50)	238.72 (24.38)
Post-nicotine	961.65 (122.13)	488.43 (54.38)	238.06 (24.14)
ANOVA (within- subj.)	F_3,54_ = 1.10,	F_3,54_ = 1.03,	F_3,54_ = 0.08,
	p = 0.36	p = 0.39	p = 0.97

Data are mean inter-response intervals (±SD) in milliseconds.

### Finger Tapping-related Neuronal Response

Finger tapping frequency did not affect BOLD response (F_3,54_ < 6, p > 0.001 uncorrected for all voxels) and all subsequent results reflect an average response across all tapping frequencies. Auditory-paced finger tapping task was associated with robust neuronal response in sensorimotor regions. In the whole-brain analysis, areas of the bilateral superior temporal gyrus (STG), left primary motor cortex (PMC), left supplementary motor area (SMA), and right superior lateral cerebellum showed response during this task (Table 2). The left STG cluster extended into the insula, striatum and thalamus; while the cerebellar activation extended into the anterior superior vermal area. There was substantial overlap between these clusters and the task-related ROIs, selected a priori, used in the subsequent analysis. 

**Table 2:** Peak neuronal responses in the whole brain analysis during auditory-paced finger tapping task, averaged across all experimental conditions.

		Coordinates:			
Region:	Volume (voxels):	x	y	z	T (max.):
L. STG	1666	-48	-22	13	15.5
R. STG	566	63	-22	4	12.9
L. PMC	272	-45	-10	55	11.48
L. SMA	124	-6	-7	61	10.8
R. cerebellum	48	24	-61	-17	9.22

All voxels thresholded at p > 0.05, FWE corrected.

STG: superior temporal gyrus.

PMC: primary motor cortex.

SMA: supplementary motor area.

The R. cerebellar cluster extended into the vermus.

There was no main effect of session (F_1,54_ < 12, p > 0.001 uncorrected for all voxels). The ANOVA interaction effect of treatment with scan order is detailed below for finger tapping-related areas as ROIs.

### The Effect of Nicotine on Finger Tapping-related Neuronal Response

**Figure 1:**
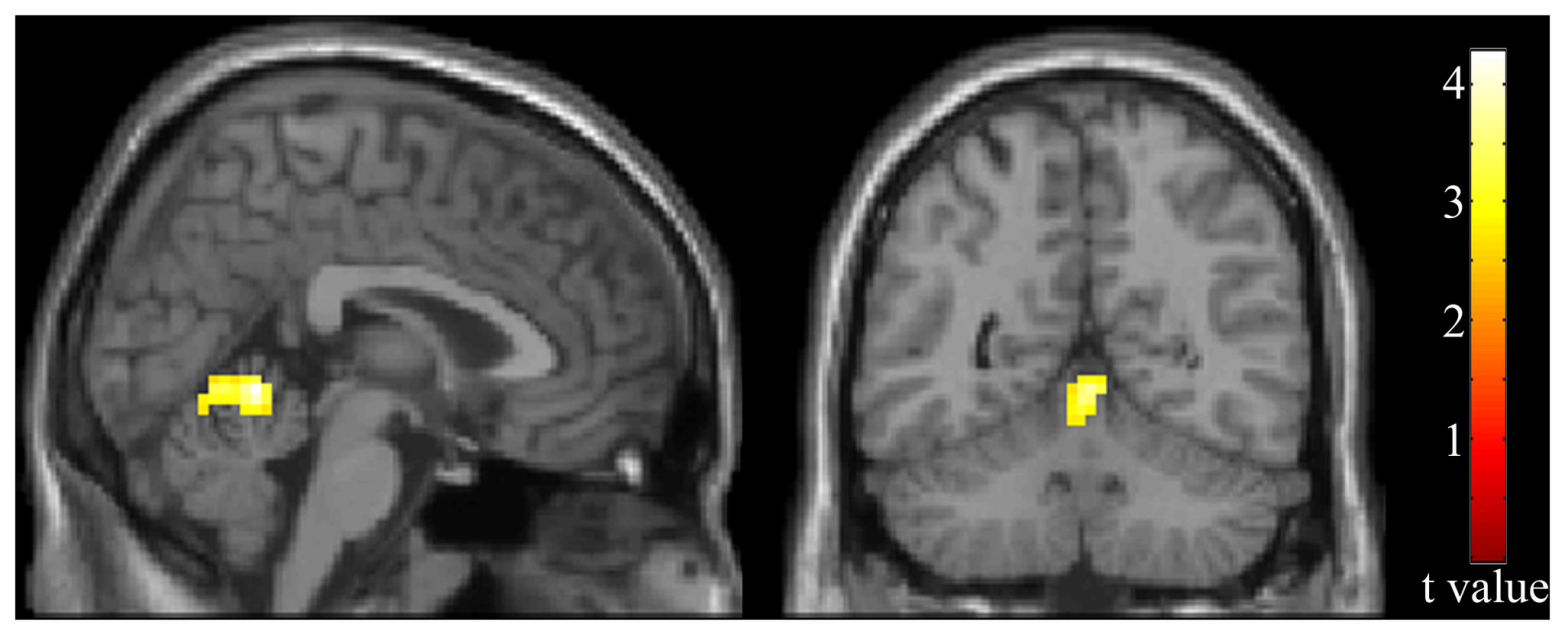
Hemodynamic response due to nicotine treatment during an auditory-paced finger tapping task. Shown is the treatment effect of nicotine in task-related ROIs, with all voxels thresholded at p < 0.01, uncorrected. Cluster was significant using small volume correction with FDR (k=54, T(max)=4.26, df=18, p=0.015).

**Figure 2:**
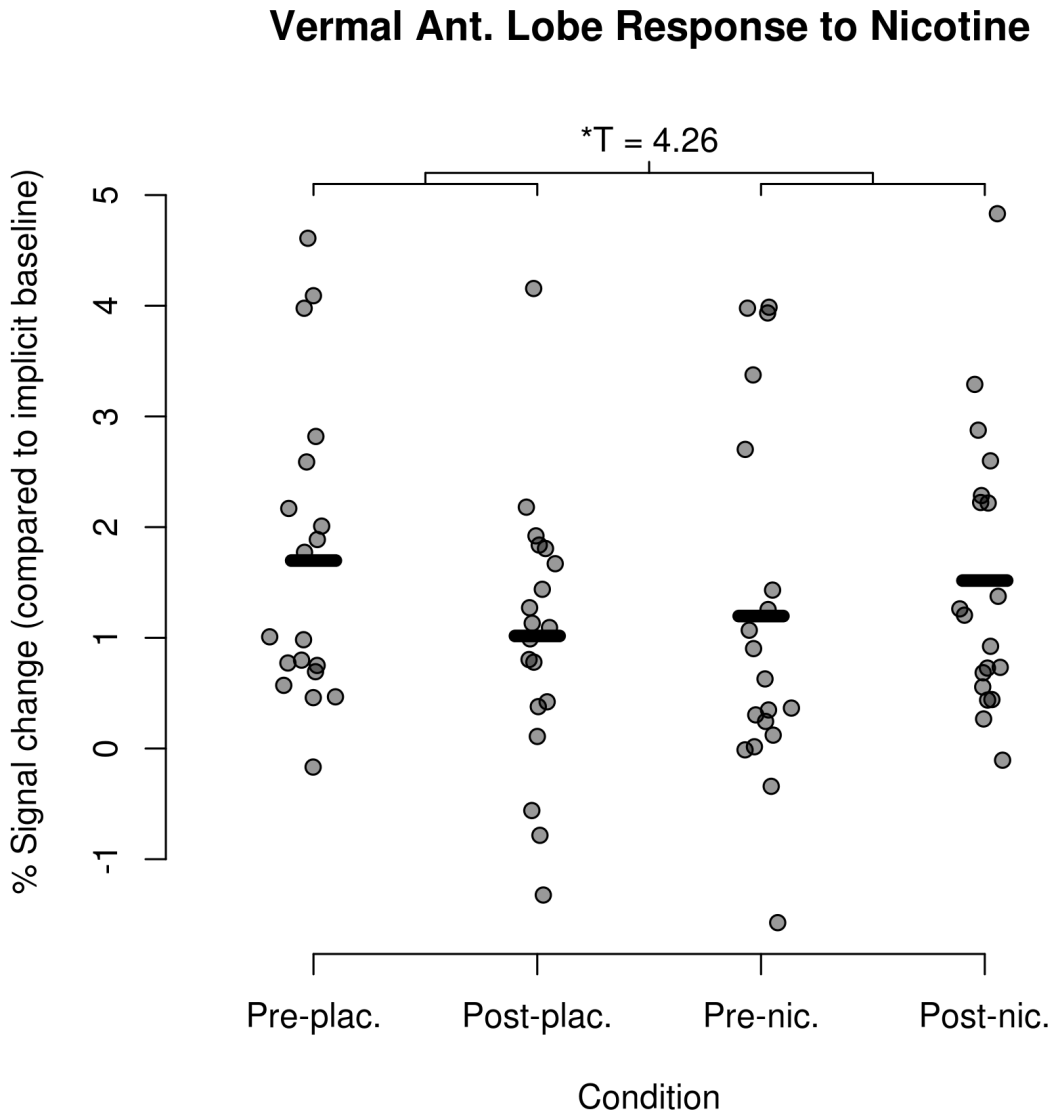
Vermal anterior lobe response to nicotine. Mean hemodynamic response by subject & condition from the peak activation within the vermal anterior lobe, with bars indicating the sample mean for each condition.

Task-related, time by drug responses in the left PMC, left SMA, right anterior cerebellar ROIs did not significantly change in response to nicotine (T < 3, df=18, p > 0.001, uncorrected for all voxels). Task-related activation in the anterior vermal cerebellum significantly increased with nicotine treatment (Figure 1, k=54, T(max)=4.26, df=18, p=0.015 FWE corrected). Anterior vermal cerebellum responses for all individuals and experimental conditions are shown in Figure 2.

## Discussion

We examined the effect of nicotine treatment on auditory-paced finger tapping in healthy subjects. Previous studies, using an unpaced finger tapping task, have shown that nicotine increases tapping rate [1]. This study, in contrast, used a paced finger tapping task and did not find an affect of nicotine on tapping rates. The performance improvement due to nicotine in unpaced finger tapping tasks could be due to either increased coordination or increased psychomotor arousal. The former effect is more amenable to conscious control and would be unchanged during a paced task. Our results, using an auditory paced finger tapping, are consistent with this and suggest that the performance enhancing effects of nicotine are due to increased coordination.

Task-related response included contralateral primary motor & SMA, bilateral STG, and ipsilateral anterior cerebellum extending into the cerebellar vermis. These areas are consistent with previously published responses using similar tasks [5-7]. The contributions of these areas to finger tapping are an area of ongoing study and have been covered, in detail, elsewhere (see for example, [5]).

Nicotine treatment increased the hemodynamic response in the vermal area of the anterior cerebellum during the task (Figure 1). Finger tapping-associated response has been reported with this area previously, most often during finger tapping tasks with moderate to high degrees of difficulty, such as tapping complex sequences or during visually paced tapping [18,19]. Simple and/or self-paced finger-tapping studies more often have found cerebellar response in the more lateral aspects of the anterior cerebellum [20]. Vermal responses to auditory-paced finger tapping have, however, been observed previously [5,21]. These responses have been suggested to stem from vermal involvement in timing-dependent activity and perception, as demonstrated by its activity during rhythmic speech [22], discrete compared to continuous finger movement [23], and judging the duration of auditory tones [24]. It is possible that, by increasing response in the vermal cerebellum, nicotine could improve timing-related coordination. This mechanism could explain nicotine’s ability to increase maximum sustainable tapping rates during unpaced finger tapping tasks [1].

These results are unlikely due to a general effect of nicotine, based on responses from other tasks. Nicotine’s effects on tasks such as sustained attention or working memory, have been shown to involve increases in parietal regions [25,26]. An additional exploratory analysis of data from the present study examining nicotine effects at a liberal whole-brain threshold of p < 0.05, uncorrected, did not reveal nicotine effects in this region. This observation suggests that previous findings of nicotine’s effects in parietal regions are likely task-specific. Nicotine also has not been shown to change regional cerebral blood flow, further suggesting that the results reported here are specific to the task in the presence of this drug, and not due to a general effect of the drug [27].

Observation of a nicotine-related increase in activity of the vermal area may reflect its effects on cerebellar Purkinje cells. In the cerebellum, Purkinje cells and mossy fibers have nAChRs and receive cholinergic afferents from other areas. Vestibular afferents project to the anterior vermal cerebellum as well as to more inferior areas such as the flocculonodular lobe [28]. Additionally, the molecular layer of the cerebellar cortex contains alpha-7 and alpha-4 nAChR on Purkinje cells [29,30]. Stimulation of these alpha-7 nAChRs by nicotine leads to glutamate release in cerebellar slices, resulting in further glutamate release [9,31]. It is possible that the increased cerebellar activity observed with nicotine treatment was related to this effect, or through interacting with receptors on Purkinje cells.

The reported results should be considered in the context of study limitations. The study was a single-blinded due to ethical concerns. The physician who applied the nicotine or placebo patches and who was present during all scans (LM) was not blind to drug condition in order to insure the safety of all subjects. Additionally, the single dose of nicotine and paced tapping task used in this study limits interpretations of the drug’s effects. Previous investigations of nicotine’s effect on finger tapping used subcutaneous or intranasal routes of drug delivery. Although the 7 mg patch dose used in this study has been shown to increase attention in healthy subjects [11], the effect of transdermal nicotine on finger tapping has not been established at this dose level. It is also possible that the effect of nicotine on task performance and neural response could be different at higher concentrations, or during self-paced finger tapping at higher rates. 

In summary, we examined the effects of nicotine on a common sensorimotor task, auditory-paced finger tapping, in healthy controls. Nicotine increased neuronal response during the task in the anterior vermal cerebellum. This finding sheds light on nicotine’s effect on coordination and performance during motor tasks and may be relevant to diseases involving cholinergic dysfunction.
